# BEV-Nexus: BEV Perception Algorithm Based on Depth Perception Enhancement and Dynamic Adaptive Fusion

**DOI:** 10.3390/s26154720

**Published:** 2026-07-25

**Authors:** Xiaona Song, Haozhe Zhang, Zhengyi Huang, Jianlin Zhao, Lijun Wang

**Affiliations:** School of Mechanical Engineering, North China University of Water Resources and Electric Power, Zhengzhou 450045, China

**Keywords:** autonomous driving, 3D object detection, multimodal fusion, LiDAR, BEV (bird’s eye view)

## Abstract

This paper proposes an improved multimodal fusion framework for 3D object detection, termed BEV-Nexus, which aims to address the issues of inaccurate depth estimation and inefficient fusion paradigms in existing image-point cloud fusion methods. We introduce a Point-Cloud-Guided Depth Prediction Network (PCGD-Net), which enhances the image branch’s depth prediction capability by embedding point cloud spatial prior, ground-truth loss constraint, and projected point cloud depth filling. Additionally, we design a Dynamic Self-adaptive Feature Fusion Module (DSF-Module), which computes multimodal feature similarity using window attention and performs weighted fusion based on self-adaptive weights, resolving alignment deviations in BEV features. Finally, we propose a Dilated Attention Enhancement Block (DAEB), which expands the receptive field through dilated convolution and integrates parameter-free attention mechanism (SimAM) for feature enhancement, ensuring efficiency while improving overall feature representation. Experimental results on nuScenes validation set show that BEV-Nexus outperforms it baseline (BEVFusion) by 1.8% mAP and 1.5% NDS. On the test set, BEV-Nexus improves mAP and NDS by 1.6% and 1.4%, respectively. Furthermore, the detection FPS remains nearly unchanged, demonstrating significant lightweight advantages.

## 1. Introduction

3D object detection is a critical task for autonomous vehicles to achieve road environment perception [1], and it is of great importance for decision-making and ensuring road safety [2]. Autonomous driving perception systems typically consist of various sensors, such as cameras, LiDAR, and millimeter-wave radar [3]. These sensors provide diverse road data while exhibiting complementary characteristics, therefore, multimodal 3D object detection algorithms have become a research hotspot [4].

Currently, mainstream multimodal fusion algorithms primarily focus on fusing multi-view camera images and LiDAR point clouds [5]. Image information provides high-resolution features with strong semantic representation capabilities, while point cloud information offers clear spatial depth expression [6]. The success of the BEVFusion [7,8] model has propelled Bird’s Eye View (BEV)-based fusion approaches into the research mainstream. BEV-based methods exhibit significant advantages, such as being friendly to multi-task learning, accommodating various modalities, and achieving geometric uniformity. For different downstream tasks, multi-task support can be realized simply by replacing the prediction head and fine-tuning, making the approach more flexible.

The core of BEVFusion lies in the LSS [9] (Lift, Splat, Shoot) technique, which provides a standard paradigm for constructing image BEV features. However, since LSS only yields a probabilistic depth distribution corresponding to image positions, the computational cost of building a feature point cloud from dense image data is extremely high. To mitigate this issue, BEVFusion [7] introduces precomputation and intermittent reduction strategies. Specifically, it first precomputes the spatial correspondence between camera feature point clouds and BEV grids to avoid substantial redundant computations, and then employs parallelized GPU calls to reduce redundant computations among BEV grids, thereby significantly improving the efficiency of BEV view transformation. Consequently, this paper takes the BEVFusion [7] model as the baseline, focusing on the research and improvement of BEV fusion methods.

Currently, BEV-based multimodal fusion detection methods face a core contradiction: the accuracy improvement brought by incorporating image data is limited, while the computational cost is extremely high. Under the experimental conditions of this paper, the baseline model BEVFusion achieves a mAP of 58.3% when using only point clouds, but this increases only to 61.4% after introducing images, yielding a performance gain of approximately 3%. However, both training memory usage and inference latency increase significantly, with computational complexity roughly doubling. This indicates that the fusion value of high-resolution image information has not been fully exploited. We attribute this to the following two main reasons:

1. The front-end LSS module is optimized solely by the final detection loss, lacking accurate spatial guidance. The inaccuracy of depth information thus diminishes the value of image features.
2. BEVFusion adopts simple concatenation and convolution to fuse multimodal features. This approach neither leverages the complementary relationships between image and point cloud features nor mitigates the misalignment issues caused by depth errors, thereby reducing the benefits of fusion.

In this paper, we propose a BEV perception algorithm named BEV-Nexus, which is based on depth perception enhancement and dynamic self-adaptive feature fusion. This algorithm significantly improves the fusion performance of the image modality. The main contributions of this paper are as follows: (1) To address the problems of inaccurate depth prediction for image features and misalignment of BEV features, we propose a Point-Cloud-Guided Depth Prediction Network (PCGD-Net). As a module-level component of BEV-Nexus, PCGD-Net primarily addresses the accuracy problem in transforming image features into BEV space. By leveraging LiDAR spatial priors, point cloud ground-truth loss constraints, and projected point cloud depth filling, this network significantly improves the accuracy of image depth estimation. (2) To tackle the issues of outdated feature fusion paradigms and low fusion accuracy, we propose a Dynamic Self-adaptive Feature Fusion Module (DSF-Module). This module leverages the similarity between image features and point cloud features. It then autonomously and dynamically performs feature fusion, alleviating the alignment deviations caused by depth estimation errors. (3) We design a Dilated Attention Enhancement Block (DAEB). This block expands the receptive field using dilated convolutions and integrates 3D parameter-free attention mechanism (SimAM [10]) to enhance the overall features. Furthermore, all the above improvements are lightweight. They introduce almost no increase in model inference time, and don’t require separate pre-training for the point cloud branch. Thus, both training efficiency and inference latency are well maintained. Experimental results on the nuScenes dataset show that, compared to the baseline BEVFusion model, BEV-Nexus improves mAP by 1.8% and NDS by 1.5%, while FPS remains almost unchanged, thus striking a balance between accuracy and efficiency.

## 2. Related Work

### 2.1. Image-to-BEV View Transformation

In autonomous driving perception systems, a surround-view camera system typically comprises five to eight cameras. However, performing view transformation on data from these multi-view cameras or explicitly aligning them with point cloud BEV presents numerous challenges, such as perspective scale transformation, lack of depth information, and occlusion. Currently, three dominant paradigms exist for this view transformation: Geometry-based Inverse Perspective Mapping (IPM), Lift-Splat-Shoot (LSS), and Transformer-based projection.

The core of IPM lies in the coordinate mapping matrix and the ground plane assumption. Specifically, it first assumes that all projected points lie on the same plane (height = 0), then computes the one-to-one correspondence between the image and the ground plane based on the intrinsic and extrinsic camera parameters, and finally performs inverse mapping to reversely derive the image pixels corresponding to the BEV grid and extract features. IPM is a purely geometric transformation method that does not rely on any training strategy, and is computationally simple and efficient.

The LSS [9] algorithm constructs image BEV features through three steps: First, it divides 3D space into D depth distribution intervals, and a frustum point cloud is constructed based on camera intrinsic and extrinsic parameters. Second, LSS separates image features into C-dimensional semantic features and D-dimensional depth features, and processes them using PyTorch’s broadcasting mechanism to obtain frustum features. Finally, leveraging the one-to-one correspondence between the frustum point cloud and frustum features, along with the dataset’s calibration data, the frustum features are transformed into vehicle coordinates and divided into corresponding BEV grids. LSS is widely adopted in 3D object detection for autonomous driving, achieving a favorable balance between computational efficiency and accuracy.

Transformer-based projection methods forgo explicit depth prediction and forward geometric transformation, instead adopting a query-driven approach to enable interaction between images and BEV representations. Specifically, these methods define reference points or object queries in the BEV plane or 3D space, and then employ deformable cross-attention mechanisms or coordinate position encoding to apply these reference points to the image plane, from which features are sampled and aggregated. This paradigm effectively circumvents the biases introduced by depth estimation, but inevitably incurs higher computational overhead.

In these methods, LSS serves as a representative bridge for view transformation, enabling explicit geometric constraints and interpretable depth distributions. The choice among these methods depends heavily on the application scenario. For production-level autonomous driving, LSS-based methods are preferred due to their deployment-friendly properties (conventional convolutions, easy inference adaptation, proven maturity)—advantages that IPM (no 3D perception) and Transformer-based methods (heavy attention overhead, difficult onboard deployment) lack. This motivates our choice of LSS-based BEVFusion as the baseline and our design of PCGD-Net to enhance its depth estimation (Section 3.1).

### 2.2. Image-Based 3D Object Detection

As shown in Table 1, image-based 3D object detection algorithms can be divided into BEV-based and non-BEV methods according to their data processing approaches.

BEV-based methods transform image features into a bird’s-eye view representation conducive to 3D detection using depth estimation algorithms or attention mechanisms. BEVDet [11] generates image BEV features via the LSS algorithm and employs a point-cloud-based detection head to achieve high-precision perception. BEVDepth [12] introduces an additional depth refinement subnetwork, which leverages ground-truth LiDAR depth information for loss supervision, significantly improving depth prediction. BEV-Former [24] avoids direct depth estimation outputs, instead, it learns implicit spatial relationships among multiple views through cross-attention, directly producing BEV features that contain implicit depth information, thereby offering greater robustness. BEVStereo [25] incorporates temporal data and simultaneously predicts depth and contextual information across multiple frames, enhancing feature representation and depth details. Fast-BEV [26] proposes precomputed position indices and an efficient BEV encoder, reducing redundant computations and improving inference speed.

Non-BEV methods often achieve efficient 3D detection through Transformer architectures. DETR [27] introduces the Transformer [28] architecture into 2D object detection, achieving end-to-end prediction outputs by generating object queries. DETR3D [13] proposes a set prediction module that migrates 3D reference points to 2D images via cross-connections between 2D and 3D spaces, performing 3D position prediction for each query. PETR [14] introduces 3D position encoding, allowing reference point information to be updated directly in 3D space, eliminating the need for cross-connections and projection computations between 2D and 3D spaces. PETR-V2 [29] incorporates a temporal modeling mechanism, leveraging multi-frame data to optimize image features and reference point generation, and uses image features to weight the position encoding, thereby improving detection accuracy. MV2D [30] first obtains 2D detection results using a 2D detector, then utilizes them to generate precise object query information, achieving more accurate localization.

### 2.3. Point-Cloud-Based 3D Object Detection

LiDAR point clouds possess precise spatial modeling capabilities. As shown in Table 1, point-cloud-based 3D object detection algorithms can be categorized into point-based methods, voxel-based methods, and other approaches [31].

Point-based methods directly extract features from point clouds. The PointNet [15,32] series pioneered direct processing of point clouds, extracting point-wise features through multi-layer perceptrons (MLPs) and addressing the issues of disorder and invariance using symmetric functions and transformation matrices. PointRCNN [16] employs PointNet++ to segment foreground points in the scene, extracts features to obtain initial proposals, and then performs local feature extraction for each proposal. Point-based methods often require downsampling, and the commonly used farthest point sampling (FPS) can lead to the loss of key points. PointRCNN++ [33] proposes a distance-based downsampling strategy that adaptively adjusts the sampling rate according to varying regional densities. 3DSSD [34] introduces a feature-based farthest point sampling algorithm (F-FPS), preserving more foreground points.

VoxelNet [17] is the pioneer of voxel-based methods. It partitions the point cloud space into regular volumetric occupancies and pools the points within each voxel to represent the features of all points in that voxel, thereby addressing the irregular data structure of point clouds. To mitigate the extra computation caused by empty voxels, SECOND [18] designs sparse voxel encoding convolution, which extracts features only from non-empty dense voxels and computes an “input-output index matrix” to map the processed dense features back to sparse features. VoxelNeXt [35] designs a fully sparse convolutional detector, replacing post-processing operations such as non-maximum suppression with spatially voxel pruning, resulting in a more concise and efficient architecture. PointPillars [36] removes the height dimension partitioning of voxels, dividing the point cloud space into pillars based solely on length and width information, achieving very fast detection speeds. PillarNeXt [37] introduces targeted refinement designs and training strategy optimizations, achieving prediction accuracy even higher than that of voxel-based methods, suggesting that fine-grained modeling of height information may be unnecessary and that investing computational resources into BEV features is more efficient.

Researchers have also proposed many other methods that achieve excellent performance. PV-RCNN [19] adopts a dual-path architecture, refining voxel-based feature extraction using key point information from point clouds. PointFormer [20] explores the application of Transformers in point-cloud-based 3D object detection, achieving better contextual representation through feature interaction between local and global regions. 3DETR [38] replicates the success of DETR, obtaining object queries through random downsampling and Fourier position encoding to realize end-to-end detection.

### 2.4. Image-Point Cloud Fusion-Based 3D Object Detection

As shown in Table 1, image-point cloud fusion-based 3D object detection algorithms can also be categorized into non-BEV and BEV methods according to their processing paradigms [39]. Among these, non-BEV approaches, represented by CMT [22] and FUTR3D [23], typically adopt Transformer architectures to accomplish the detection task. They generate reference points in 3D space and transform them into different coordinate spaces, using these points as initial anchors for detection. The advantage of this approach lies in its end-to-end detection capability and the absence of explicit feature transformation. However, it suffers from relatively poor multi-task friendliness and flexibility.

BEVFusion [7,8] is a pioneering work among BEV-based methods. It transforms image features into the same BEV space as point clouds using the LSS algorithm and fuses them via concatenation, allowing seamless integration with point cloud detectors and excellent support for various downstream tasks. Transfusion [40] establishes a “soft association” between image features and point cloud features through cross-attention, yielding a more robust fused representation. FusionFormer [41] also adopts cross-attention for BEV feature fusion and introduces a BEV memory bank mechanism, further enhancing the fused features through temporal information. SparseFusion [21] argues that the dense-to-dense BEV feature fusion employed by BEVFusion is redundant and proposes a sparse-to-sparse fusion approach, which first obtains multimodal candidates via separate detectors and then performs feature fusion, significantly improving computational efficiency. IS-Fusion [42] adopts a dense-dense-sparse fusion paradigm, first obtaining instance features through scene-level fusion and then performing instance-level fusion to enhance the fused features at different granularities. In addition, several models improve performance by enhancing the depth prediction capability of the LSS algorithm. EA-LSS [43] computes depth maps and edge maps from point cloud projections and uses them to augment image branch features, thereby improving the spatial representation capability of image features. SimpleBEV [44] directly fills projected point cloud depth into the image-predicted depth, intuitively boosting depth prediction accuracy.

At present, BEV-based fusion detection algorithms still face the challenges of high computational cost and limited performance gains from fusion. A twofold increase in computational load and memory usage often yields only a 2–3% improvement in accuracy. This paper posits that inaccurate image depth estimation in LSS and outdated fusion paradigms are the primary causes of this issue. To address these problems, we propose a Point-Cloud-Guided Depth Prediction Network (PCGD-Net) and a Dynamic Self-adaptive Feature Fusion Module (DSF-Module). The former enhances the network’s depth prediction capability, while the latter fuses multimodal features more accurately and efficiently, improving the performance gain brought by the image fusion branch without sacrificing inference speed. Furthermore, we design a Dilated Attention Enhancement Block (DAEB) to further refine the fused features.

## 3. Method

**Figure 1:**
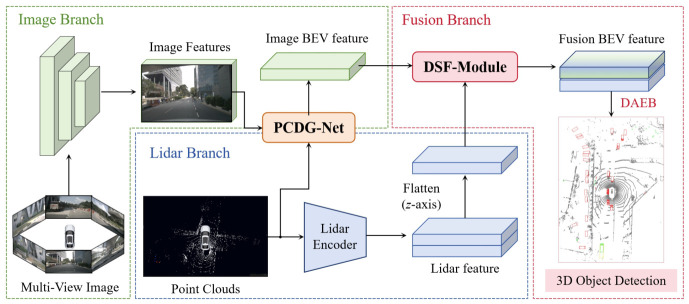
The figure illustrates the overall architecture of BEV-Nexus, which can be divided into an image branch, a Lidar branch, and a fusion branch. The image branch and Lidar branch interact and fuse through PCGD-Net and DSF-Module, and are finally enhanced by DAEB for detection.

As illustrated in Figure 1, this paper presents the proposed BEV-Nexus model, which achieves deeper and more intelligent feature interaction based on the baseline BEVFusion. This section provides a detailed introduction to its overall architecture and functional implementation: First, image features and point cloud BEV features are extracted via an image backbone network and a voxel encoding network, respectively. Then, the image features and raw point cloud data are jointly fed into the Point-Cloud-Guided Depth Prediction Network (PCGD-Net) to obtain image BEV features. Next, the image BEV features and point cloud BEV features undergo feature fusion through the Dynamic Self-adaptive Feature Fusion Module (DSF-Module) to produce multimodal fused features. Finally, after being processed by the Dilated Attention Enhancement Block (DAEB), the fused features are passed to a Transfusion-based detection head for object detection.

### 3.1. Point-Cloud-Guided Depth Prediction Network (PCGD-Net)

The BEVFusion model unifies multimodal data into the BEV perspective for prediction, and its image branch follows the basic LSS algorithm, which does not fully utilize the spatial information from point clouds. For multimodal data input, point clouds intuitively reflect the true 3D coordinates of each object, thereby providing inherent three-dimensional depth information, which can assist image-based LSS in obtaining more accurate depth estimation. The success of methods such as BEVDepth [12], EA-LSS [43] and SimpleBEV [44] demonstrates the positive effect of point cloud supervision and direct integration on improving image depth prediction performance. However, they still suffer from issues such as insufficient fusion, incomplete information utilization, and complex fusion pipelines. Building on this insight, we design a module-level component, named Point-Cloud-Guided Depth Prediction Network (PCGD-Net), which further enhances the utility of point cloud spatial information in the image branch, while maintaining computational efficiency through a lightweight design.

**Figure 2:**
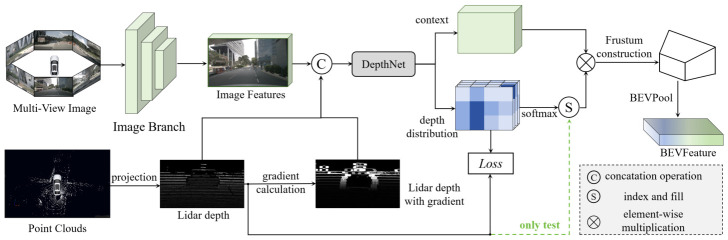
The overall structure of PCGD-Net. The point cloud is first projected into multi-view depth maps, which are then processed via gradient computation and injected into the image branch as prior information. The raw depth maps are used to compute the loss function for optimizing the depth network of the image branch. Finally, sparse projected point cloud depth is filled into the predicted depth in test phase.

PCGD-Net abandons the more computationally expensive approaches of semantic-depth separation fusion and attention-weighted fusion, instead adopting an intuitive prior injection and a concise classification loss strategy, thereby achieving a lightweight network pipeline design. The main function of PCGD-Net is to integrate point cloud depth information into the depth prediction network for image features, while simultaneously applying loss constraints to the image depth prediction results using ground-truth point clouds. This enhances the representational capacity of images while optimizing the accuracy of predicted depth. As shown in Figure 2, the network improves the performance of the LSS algorithm through three key modifications:

1. Integrating LiDAR point cloud depth information into the image feature representation, thereby providing spatial prior information for image depth prediction.
2. Constraining the image-predicted depth using LiDAR point clouds, enabling finer optimization of the depth prediction network during training.
3. During the testing phase, we introduces the projected point cloud depth to further refine depth representation.

First, PCGD-Net projects the point cloud into the multi-view image coordinate system via coordinate transformation and removes points that fall outside the image boundaries, obtaining the projected point cloud depth at corresponding image positions. However, due to the sparsity of LiDAR point clouds, the resulting depth map is highly sparse and contains numerous zero-valued regions, which leads to suboptimal integration into image features.

To mitigate this issue, we first applies a sliding-window-based local max-pooling operation to the sparse point cloud depth map, extending the valid projected points to their surrounding regions. This step makes the depth distribution denser and smoother while stabilizing the gradients. Subsequently, the depth map is shifted in four directions (up, down, left, right) with a stride equal to that of the local max-pooling, and the differences (gradients) between the shifted and original depth maps are computed. Next, the gradient tensors from the four directions are concatenated with the smoothed depth map to obtain a depth representation that contains both depth information and gradients. Finally, this representation and the sparse depth map are fused into the image features, thereby injecting depth prior information into the image branch and facilitating the training of the subsequent depth prediction network.

Next, image features are extracted by the depth prediction network and then split to obtain C-dimensional semantic features and D-dimensional depth features. In the basic LSS algorithm, after this step, the depth features are normalized and used to construct frustum features via an outer product with the semantic features, a process also adopted by the baseline BEVFusion. However, this approach can only back-propagate and optimize parameters through the loss function of the final object detection task. Due to the complex parameter composition and gradient paths in this process, the depth prediction network itself struggles to receive sufficient and targeted training, limiting improvements in depth prediction performance.

To address this issue, we introduces a separate depth supervision loss function, aiming to directly supervise the depth prediction network using ground-truth point cloud depth. Specifically, PCGD-Net has already projected the point cloud into the multi-view image coordinate system and obtained multi-view ground-truth depth maps. This paper defines the detection depth range and intervals as: near = 1 m, far = 60 m, interval = 0.5 m, thus dividing the 3D space into 118 depth intervals. Then, projected points falling outside the range are discarded, and the depth values of the remaining points are discretized into these 118 intervals. Consequently, the loss computation of the predicted depth probability distribution is converted into a classification loss over 118 “categories”, we introduced Focal Loss [45] to process it, expressed by the following formulas:

(1) $$\begin{array}{c} Mask\left(i\right)=\left\{\begin{array}{cc} 1 & 1\leq D_{Lidar}\left(i\right)<60\\ 0 & otherwise \end{array}\right.\;V=\left\{i|Mask\left(i\right)=1\right\} \end{array}$$

(2) $$\begin{array}{c} D_{class}\left(i\right)=\left\lfloor \frac{D_{Lidar}\left(i\right)-1}{0.5}\right\rfloor \in \left\{0,1,\ldots ,118\right\},\;i\in V \end{array}$$

(3) $$\begin{array}{c} Loss_{depth}=-\frac{1}{N}\sum_{i=1}^{N}\alpha {\left(1-\sigma \left(D_{pre}\left(i,D_{class}\left(i\right)\right)\right)\right)}^{\gamma }\log\left(\sigma \left(D_{pre}\left(i,D_{class}\left(i\right)\right)\right)\right) \end{array}$$

In this formulas: $D_{Lidar}\left(i\right)$ denotes the depth of the ground-truth point cloud; Mask(i) and *V* are used to create position mask indices, which discard points falling outside the valid range while using i∈V to ensure that the loss is computed only at positions where the projected point cloud depth has valid values; $D_{class}\left(i\right)$ is the discretized class representation corresponding to the point cloud ground-truth depth, obtained by dividing $D_{Lidar}\left(i\right)$ into 118 depth intervals with a spacing of 0.5 m; *N* is the number of valid pixels (those within the depth range and with valid depth values), used for loss normalization; σ is the sigmoid normalization function, which maps the predicted depth to a normalized probability; $\sigma (D_{pre}\left(i,D_{class}\left(i\right)\right))$ denotes the normalized probability of the predicted depth for the i-th pixel, which is used to compute the focal loss, where $D_{class}\left(i\right)$ filters the class channels to compute the loss only for the ground-truth depth class; When the predicted probability $\sigma (D_{pre}\left(i,D_{class}\left(i\right)\right))$ is close to 1, the sample is easier to classify, and the $(1-\sigma \left(D_{\mathrm{pre}}\left(i,D_{class}\left(i\right)\right)\right)$ becomes smaller; By setting the modulating factor γ (here set to 2), its contribution can be reduced, making the model focuses more on difficult samples; α (set to 0.25) suppresses the interference from a large number of negative samples, helping to maintain the balance between positive and negative samples.

Finally, PCGD-Net introduces projected point cloud point cloud depth as a supplement to the final depth information before frustum feature construction. To avoid affecting network training, this step is performed only during test phase. Due to the sparsity of the point cloud projection view, PCGD-Net performs range filtering, position masking, and one-hot encoding to fill depth data at positions where projected point cloud point cloud projections exist with the true depth values, while using the depth prediction network’s predicted depth elsewhere. This step is intended to further refine and complement the results of the image depth prediction branch, while ensuring the model’s robustness to deviations in the prediction network under unexpected circumstances. The masking and filling operations involved do not introduce any additional computational overhead.

PCGD-Net improves depth prediction performance while maintaining an efficient lightweight design. During model inference, it only adds basic projection computations (coordinate transformation, perspective projection, boundary checking, and assignment), gradient computation, and projected point cloud depth filling operations (masking, indexing), resulting in negligible computational overhead.

### 3.2. Dynamic Self-Adaptive Feature Fusion Module (DSF-Module)

After obtaining the image and point cloud BEV features respectively, BEVFusion employs channel concatenation followed by 3 × 3 convolution for further fusion. However, this fusion approach suffers from low-level integration and poor robustness, potentially amplifying errors from BEV transformation. Models such as Transfusion and FusionFormer adopt attention mechanisms for multimodal feature fusion, avoiding BEV transformation errors and feature alignment issues, demonstrating the effectiveness of adaptive weights in improving fusion efficiency. However, their primary purpose is to fuse multimodal features for 3D object detection, and they therefore employ high-dimensional global self-attention and cross-attention, combined with multi-layer residual connections and multi-stage fusion strategies, which introduce significant computational overhead. Inspired by these methods and taking into account the application scenario of this paper, we design a lightweight Dynamic Self-adaptive Feature Fusion Module (DSF-Module), which aims to perform adaptive dynamic feature fusion based on the similarity between image and point cloud features, thereby avoiding a series of problems associated with static fusion.

**Figure 3:**
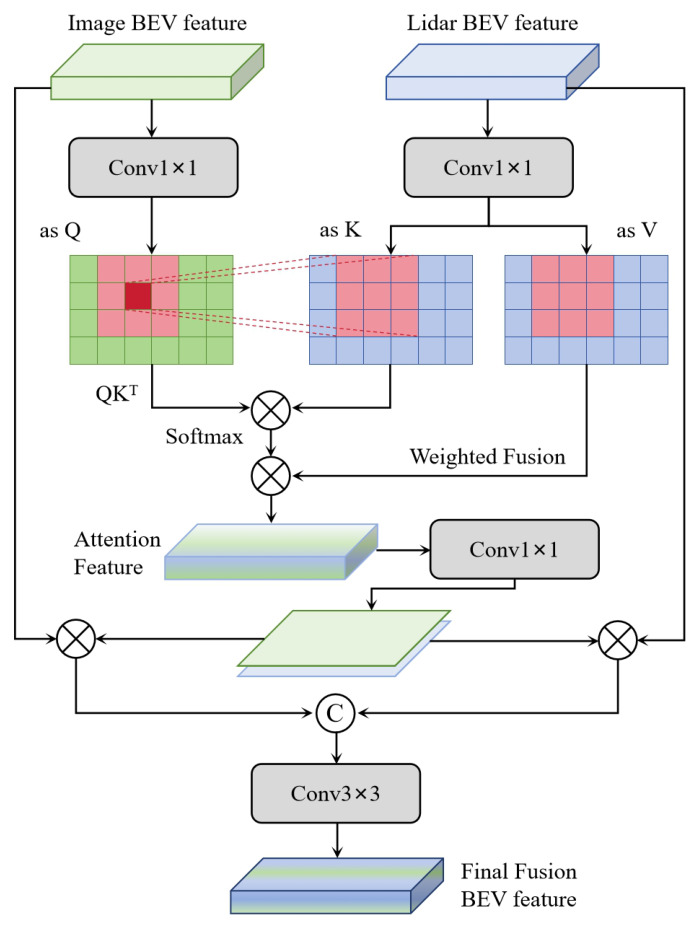
The overall structure of DSF-Module. The image features and point cloud features are first compressed in dimensionality for window attention computation. The resulting attention features are then compressed into two channels via convolution, which are used to dynamically and adaptively weight the multimodal features.

As shown in Figure 3, the module first compresses the channels of the image features and point cloud features using 1 × 1 convolutions. The image features are transformed into 64 dimensions to facilitate subsequent attention computation, while the point cloud features are compressed to 128 dimensions and then split into two 64-dimensional features. This design balances the trade-off between information density and computational efficiency, allows feature computation to remain at a higher spatial dimensionality under the same computational budget, which benefits network training.

Next, the module performs attention computation with the image features serving as Query and the point cloud features as Key and Value, aiming to capture the similarity between the two modalities. However, global attention would introduce significant computational overhead. Taking a 90 × 90 feature map and an attention dimension of 64 as an example, even a single-head global attention would add a computational complexity of approximately $2N^{2}D^{2}$ (where N = 90 × 90 and D = 64). Therefore, this paper adopts window attention for feature fusion: First, the image BEV features and point cloud BEV features are spatially divided into 225 windows of size 6 × 6 (corresponding to a physical receptive field of 7.2m×7.2m, which is sufficient to capture relevant features). Then, cross-attention is computed only between windows at the same spatial locations, local interaction between image and point cloud features is performed within each window, without any cross-window information propagation. This significantly reduces the computational complexity of global attention while satisfying the requirements of DSF-Module. Compared with global attention, Window attention reduces the computational complexity to approximately $2MS^{2}D^{2}$ (where M is the number of windows and S is the window size), achieving a reduction factor of about 225×.

Moreover, window attention does not cause significant accuracy degradation. This is because the fusion operation essentially computes a similarity matrix between image features and point cloud features within the same local region, rather than performing long-range modeling within a single modality. Consequently, it does not rely on the broad receptive field of global attention but focuses more on local neighborhood information matching.

Finally, after window attention computation, the module generates multimodal attention features that incorporate weighted interaction information between the spatial and channel dimensions of the image and point cloud features. Subsequently, a 1 × 1 convolutional layer compresses the attention features into a two-channel weight map, which is used to perform weighted fusion of the original image features and point cloud features, ultimately producing the fused BEV features.

Through the above processing, the image features and point cloud features achieve a dynamic and adaptive fusion process, which avoids the issues of low-level fusion, lack of dynamic adaptability, and poor robustness in concatenation-based methods, significantly improving the model’s feature representation. This paper analyzes the parameter count and computational complexity of the module, demonstrating its advantages in terms of lightweight design (with a fixed BEV feature map size of 90 × 90):

1. Compared with the fusion method of BEVFusion, DSF-Module mainly adds three 1 × 1 convolutions in terms of parameter count: one to compress the 80-dimensional image features to 64 dimensions, one to compress the 256-dimensional point cloud features to 128 dimensions, and one to compress the 64-dimensional attention features to 2 dimensions. According to the formula for convolutional parameter calculation (Equation (4)), the additional parameters amount to approximately 38 k.

2. In terms of computational complexity, besides the 1 × 1 convolution operations, the window attention computation and feature weighting are also added. According to Equations (5) and (6) (a full attention computation requires two evaluations of Equation (6)), the computational cost excluding weighting is approximately 0.34 G MACs (Multiply-Accumulate operations). Since the weighting operation consists only of multiplications (element-wise multiplication), its cost is about 0.0027 GFlops, which is negligible given that 1 MAC typically corresponds to 2 Flops.

3. Using the concatenation plus 3 × 3 convolution of BEVFusion as the baseline, which compresses 336-dimensional features to 256 dimensions, the parameter count and computational complexity are approximately 774 k and 6.27 G MACs, respectively.

(4) $$\begin{array}{c} Params_{\mathrm{conv}}=C_{in}\cdot C_{out}\cdot K^{2} \end{array}$$

(5) $$\begin{array}{c} MACs_{\mathrm{conv}}=H\cdot W\cdot C_{in}\cdot C_{out}\cdot K^{2} \end{array}$$

(6) $$\begin{array}{c} MACs_{\mathrm{window}\_\mathrm{attn}}=M\cdot S^{2}\cdot D \end{array}$$

In these formulas: $C_{in}$ and $C_{out}$ denote the number of input and output channels of the convolutional layer, respectively; *K* is the convolution kernel size; *H* and *W* are the height and width of the feature map; *M* is the number of windows in window attention; *S* is the window size; and *D* is the attention dimension.

In conclusion, while DSF-Module enhances multimodal feature fusion, it introduces only approximately 4.8% more parameters and 5.4% higher computational overhead compared to a 3 × 3 convolution in the baseline model. These increases are negligible for the overall model, showcasing significant lightweight advantages.

### 3.3. Dilated Attention Enhancement Block (DAEB)

Spatial attention or channel attention are common strategies used by researchers to improve feature representation. These methods weight the input features by computing attention weights along the channel or spatial dimensions, enabling the model to focus more on important features. The most typical attention enhancement modules, such as CAM [46], SE-Net [47], and CBAM [46], have demonstrated their effectiveness in improving model performance. However, due to computational complexity constraints and the combined use of 1D and 2D attention mechanisms, they suffer from limited receptive fields and suboptimal weighting of 3D features. To address these issues, this paper proposes the Dilated Attention Enhancement Block (DAEB), which combines dilated convolution with a parameter-free 3D attention mechanism (SimAM [10]), achieving better performance while maintaining efficiency.

**Figure 4:**
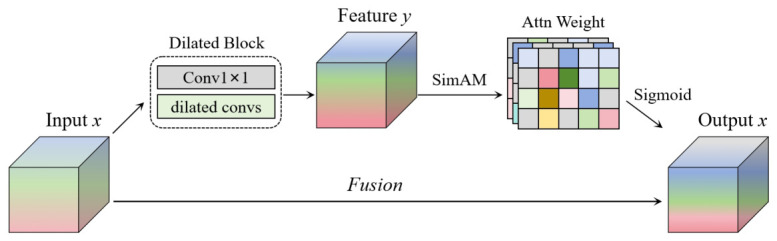
The figure illustrates the overall structure of DAEB. First, the original features are passed through a dilated convolution block to expand the receptive field, and then SimAM is applied to compute 3D attention weights, which are multiplied with the original features to achieve feature enhancement.

As shown in Figure 4, unlike conventional channel or spatial attention mechanisms, the attention weights in DAEB are generated from a dilated convolution branch of the input features rather than from the input features themselves. The introduction of the dilated convolution branch ensures that the weight generation focuses not only on the object itself but also incorporates rich contextual and background information. By combining dilated convolution with SimAM to obtain the attention weights, the module can achieve a larger receptive field and capture more surrounding environmental features.

SimAM adopts a parameter-free design and is a genuine 3D attention mechanism, rather than a concatenated combination of 1D and 2D attention. SimAM reformulates the logic of attention weight generation, replacing pooling operations with an energy function while eliminating a series of operations such as fully connected layers, thereby achieving the generation of 3D weights. SimAM first computes the energy of each neuron to evaluate its importance. According to the theory of spatial suppression, lower energy indicates that the neuron is more distinct from its surroundings, and thus the attention score should be higher. SimAM adopts a binary classification idea to represent the energy difference between the target neuron and other neurons. Through a linear function (wx+b), it drives other neurons to approach −1 and the target neuron to approach 1. The formulas can be expressed as follows:

(7) $$\begin{array}{c} e_{i}\left(w_{t},b_{t},y,x_{i}\right)=\frac{1}{M-1}\sum_{i=1}^{M-1}\left({\left(-1-\left(w_{t}x_{i}+b_{t}\right)\right)}^{2}+{\left(1-\left(w_{t}t+b_{t}\right)\right)}^{2}\right)+\lambda w_{t}^{2} \end{array}$$

(8) $$\begin{array}{c} w_{t}=-\frac{2(t-\mu_{t})}{{\left(t-\mu_{t}\right)}^{2}+2\sigma_{t}^{2}+2\lambda },\;b_{t}=-\frac{1}{2}\left(t+\mu_{t}\right)w_{t} \end{array}$$

In these formulas, *t* denotes the current spatial location of interest (the t-th pixel); *M* represents the total number of spatial locations in the feature map (H×W); $x_{i}$ denotes all neurons in the same channel except *t*; $w_{t}$ and $b_{t}$ are the weight and bias for neuron *t*, computed according to Equation (8), which is derived by taking the partial derivatives of the energy function with respect to $w_{t}$ and $b_{t}$ in order to minimize the energy; For neurons with higher discriminability, $\left(w_{t}x_{i}+b_{t}\right)$ should be closer to −1, and $\left(w_{t}x_{t}+b_{t}\right)$ should be closer to 1, thus making the ${\left(-1-\left(w_{t}x_{i}+b_{t}\right)\right)}^{2}$ and ${\left(1-\left(w_{t}t+b_{t}\right)\right)}^{2}$ smaller and resulting in lower neuron energy; λ (set to $1\times e^{-4})$) is a balancing coefficient, which is used to reduce the numerical impact caused by weight variations and to prevent overfitting; and $\mu_{t}$ and $\sigma_{t}$ are the mean and variance of all neurons except *t*, respectively.

After computing the energy of each neuron, SimAM applies a sigmoid normalization to its reciprocal and multiplies it with the features for enhancement, outperforming traditional channel attention mechanisms in both performance and flexibility. By integrating the advantages of dilated convolution and SimAM, DAEB can further improve the model’s detection accuracy at very low cost.

## 4. Experiments

### 4.1. Datasets and Evaluation Metrics

In this paper, we conduct comparative experimental validation on the nuScenes dataset [48]. The nuScenes dataset is a large-scale open-source multimodal dataset in the field of autonomous driving object detection, released by the nuTonomy team in 2019. It consists of 1000 scenes, each containing 20 s of video recordings. Image and point cloud data are captured using six surround-view cameras and one 32-beam LiDAR, respectively, resulting in up to 1.4 million images and approximately 400,000 frames of point cloud data. The dataset provides 1.4 million 3D bounding boxes across 23 fine-grained categories, supporting a variety of environment perception tasks.

We adopt the official nuScenes evaluation metrics for performance assessment, which mainly include: mean Average Precision (mAP), nuScenes Detection Score (NDS), mean Average Translation Error (mATE), mean Average Scale Error (mASE), mean Average Orientation Error (mAOE), mean Average Velocity Error (mAVE), and mean Average Attribute Error (mAAE). Among these, mAP and NDS represent the overall detection accuracy of the model, while mATE, mASE, mAOE, mAVE, and mAAE reflect the model’s performance in other aspects.

### 4.2. Implementation Details

In this paper, the backbone network selection follows the configuration of the baseline BEVFusion model. However, due to GPU resource limitations (a single RTX 4090), we are unable to conduct experiments at full resolution. Therefore, to ensure a fair comparison under identical settings, we uniformly rescale the resolution, reducing the BEV feature map size from 180 × 180 to 90 × 90, i.e., to one-quarter of the original. For the point cloud branch, VoxelNet is employed as the backbone, with the voxel size set to 0.15 m (compared to 0.075 m in the original BEVFusion), a scaling factor of 8, and FPN as the neck network. The image backbone adopts Swin-T with an input resolution of 704 × 256, and the neck network employs FPN, with a 4× downsampling applied to the image features to match the BEV feature size.

During training, the point cloud branch adopts the following data augmentation strategies: global rotation (rotation angle range [−π/4, π/4]), global scaling (scale range [0.9, 1.1]), global translation (standard deviation of 0.5 m), random flipping (implemented via BEVFusionRandomFlip3D), as well as class-balanced sampling (implemented via the CBGSDataset wrapper) and database sampling to mitigate class imbalance issues. The image branch is augmented with the same global rotation, global scaling, and global translation. The loss function consists of four components: a heatmap loss (GaussianFocalLoss, weight 1.0), a classification loss (Focal Loss, weight 1.0), a bounding box regression loss (L1 Loss, weight 0.25), and the depth supervision loss from the PCGD module (Focal Loss, weight 1.0). The total loss during training is the weighted sum of these four components.

All experiments are conducted on a single NVIDIA RTX 4090 GPU (24 GB VRAM). The software environment consists of Ubuntu 20.04, PyTorch 1.10.0, Torchvision 0.11.0, CUDA 11.3, and cuDNN 8.6, with the implementation based on the MMDetection3D v1.3.0 codebase. The training seed is set to 309315180. For inference speed evaluation, we report the average frame rate (FPS) measured with a batch size of 1 after 50 warm-up iterations. It should be noted that the projected point cloud depth filling operation via point cloud projection during the testing phase is performed during inference, and its runtime has been accounted for in the reported FPS, ensuring a fair comparison with other methods under identical hardware conditions.

The training process of the BEV-Nexus model consists of two stages: First, the point cloud network is trained independently with a batch size of 4, an initial learning rate of $1.25\times 10^{-5}$, using the AdamW optimizer and a cyclic learning rate schedule, for a total of 20 epochs. Subsequently, the image branch is added for joint training. The image branch uses Swin-T pre-trained weights from ImageNet-1k, with a learning rate set to $2\times 10^{-5}$. After merging, joint training is performed for 6 epochs.

### 4.3. Main Results

To validate the effectiveness of the proposed improvements, we reproduced the baseline BEVFusion model under identical experimental settings (voxelization size, training batch size, feature dimensions, etc.) for a detailed comparison. As shown in Table 2, the BEVFusion baseline achieves an mAP of 61.4% and an NDS of 66.1%, which are comparable to other methods (e.g., FUTR3D, Transfusion). Under the same experimental environment and parameter configuration, the proposed BEV-Nexus model attains an mAP of 63.2% and an NDS of 67.6%, improving mAP and NDS by 1.8% and 1.5% over the baseline, respectively. Furthermore, on the test set, our proposed BEV-Nexus model improves mAP and NDS by 1.6% and 1.4%, respectively, significantly enhancing detection accuracy. This section also compares BEV-Nexus with other contemporaneous models under the same resolution, demonstrating that BEV-Nexus maintains a notable performance advantage.

Subsequently, we compare the per-class average precision of BEV-Nexus and the BEVFusion baseline on the nuScenes dataset in Table 3. It can be observed that BEV-Nexus achieves consistent performance improvements across all categories. Notably, on the baseline’s weaker categories such as construction vehicles and barriers, our method improves the average precision by 4.0% and 5.3%, respectively, demonstrating the effectiveness of our approach in enhancing the overall fused feature representation of the network.

Furthermore, in Table 4, we present experimental results on the mATE, mASE, mAOE, mAVE, and mAAE metrics. BEV-Nexus also demonstrates consistent accuracy improvements: except for a slight increase of 0.1% in mAAE, all other error metrics decrease. Notably, mAOE and mAVE are reduced by 3.9% and 1.2%, respectively, demonstrating that our proposed model significantly improves orientation localization and velocity prediction accuracy.

Model’s complexity is another important aspect for evaluating model effectiveness and practicality. Since the improvements proposed in this paper over the BEVFusion model all exhibit significant lightweight advantages, BEV-Nexus also performs excellently in terms of efficiency and scale. As shown in Table 5, it can be observed that while achieving substantial improvements in detection performance, BEV-Nexus maintains memory usage and inference speed comparable to the baseline method. This result is attributed to the various lightweight designs in our work: the total parameters introduced by PCGD-Net and DSF-Module are less than those of a single 3 × 3 convolution with 256 channels, and DAEB ensures efficiency through dilated convolution and a parameter-free attention mechanism. Furthermore, BEV-Nexus also employs sparse convolution to improve runtime efficiency.

**Figure 5:**
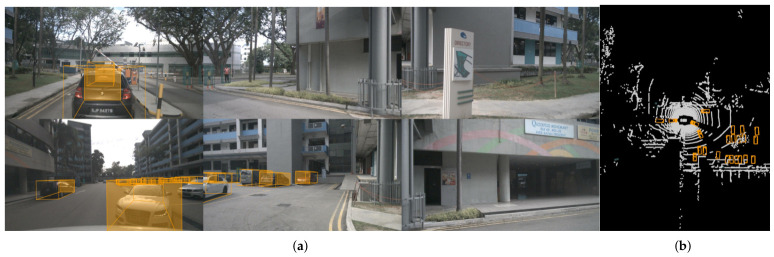
Visualization of detection results on the nuScenes dataset. Figure (**a**) shows the detection results on multi-view images, and Figure (**b**) shows the detection results on LiDAR point clouds.

Finally, we visually demonstrate the object detection performance of the BEV-Nexus model through qualitative results. Figure 5 presents an overview of the detection capability of BEV-Nexus, showing that it accurately detects valid targets in the scene, achieving excellent detection performance.

**Figure 6:**
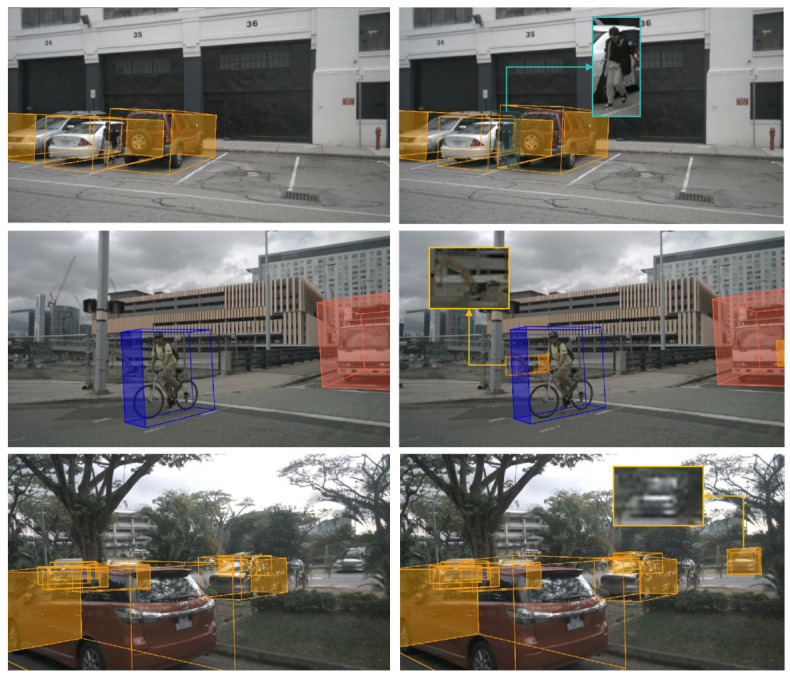
Visualization comparison with the baseline method. The **left** shows the detection results of the baseline, and the **right** shows the detection results of BEV-Nexus.

In Figure 6, we further provide a detailed comparison of the detection details between the BEVFusion model (left) and the BEV-Nexus model (right). Analyzing the images reveals the following: In the first scene, a pedestrian stands among several vehicles. The BEVFusion model struggles to distinguish the pedestrian, resulting in a missed detection, while BEV-Nexus accurately detects the pedestrian occluded by vehicles. In the second scene, which depicts a construction zone, a construction vehicle is simultaneously occluded by a cyclist and a barrier. Moreover, the construction vehicle category has few samples in the nuScenes dataset, leading to a missed detection by BEVFusion. In contrast, BEV-Nexus successfully identifies the target in this challenging scenario. In the third scene, a complex road section with abundant greenery and many vehicles, BEVFusion accurately localizes most vehicles but misses a object occluded by vegetation, whereas BEV-Nexus precisely detects every vehicle in the scene. These results demonstrate that BEV-Nexus achieves better detection performance across multiple scenarios, validating the effectiveness of our improvements over the baseline model.

### 4.4. Ablation Experiment

In this section, we conduct an ablation study on the proposed improvements. Table 6 presents the overall ablation results of the model. The results show that PCGD-Net improves mAP and NDS by 0.8% and 0.4%, respectively. Adding DSF-Module further improves mAP and NDS by 0.6% and 0.3%, respectively. The introduction of DAEB further increases mAP and NDS by 0.4% and 0.8%, respectively.

We further analyze the synergistic relationships among the three modules. When PCGD-Net and DSF-Module are combined, the mAP improves by 1.4% over the baseline, which exceeds the sum of their individual gains (0.8% + 0.3% = 1.1%). This indicates that the enhanced depth information provided by PCGD-Net offers more reliable spatial geometric guidance for the cross-modal attention in DSF-Module. When all three improvements are incorporated, mAP and NDS improve by 1.8% and 1.5%, respectively, again exceeding the sum of their individual gains (mAP: 0.8% + 0.3% + 0.3% = 1.4%; NDS: 0.4% + 0.2% + 0.6% = 1.2%), with excesses of approximately 0.4% and 0.3%, respectively. This demonstrates a clear synergistic effect among the three modules—PCGD-Net improves depth estimation quality, DSF-Module builds upon this to generate more accurate fusion weights, and DAEB further expands the receptive field and recalibrates channels of the fused features. The combined benefit is not a mere superposition of individual performance gains. Subsequently, we perform further ablation and visual validation on the details of these improvements.

#### 4.4.1. Ablation Study on PCGD-Net

**Figure 7:**
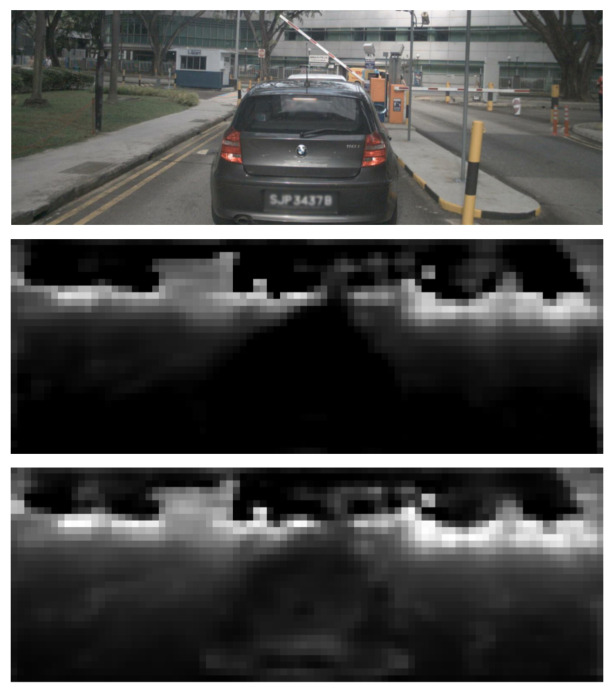
Visualization comparison of depth prediction performance. The **top** row shows the original images, the **middle** row shows the depth maps predicted by the baseline model, and the **bottom** row shows the depth maps predicted by the BEV-Nexus model.

As shown in Figure 7, we qualitatively analyze the improvement of PCGD-Net in depth prediction accuracy from a visual perspective. The top row shows the original scene images, the middle row shows the depth maps predicted without PCGD-Net, and the bottom row shows the depth maps predicted after introducing PCGD-Net. It can be observed that the original depth maps can only roughly separate the depth intervals of the foreground and background, with uneven depth distribution and difficulty in distinguishing foreground objects. In contrast, after introducing PCGD-Net to assist depth network prediction, the contours of foreground objects are delineated more clearly, which is beneficial for improving detection performance.

Subsequently, we quantitatively analyze in Table 7 the performance improvements brought by each of the three modifications in PCGD-Net: point cloud spatial prior, ground-truth loss constraint, and projected point cloud depth filling. The experimental results show that the introduction of the point cloud spatial prior and the ground-truth loss constraint contribute the most to the accuracy gains, improving mAP by 0.7% and NDS by 0.4%, respectively, thereby significantly enhancing depth prediction accuracy. When projected point cloud depth filling is used alone, mAP and NDS increase by 0.4% and 0.2%, respectively. However, when combined with the first two improvements, the additional gain in mAP is reduced to 0.1%. Therefore, the introduction of the point cloud spatial prior and the ground-truth loss constraint already enables the depth prediction network to adequately learn the true depth distribution, while projected point cloud depth filling serves as an auxiliary means to further refine depth representation, thereby guaranteeing the lower bound of depth estimation performance.

#### 4.4.2. Ablation Study on DSF-Module

As shown in Table 8, we conduct an ablation study analysis on the DSF-Module, mainly comparing it with the basic convolution scheme. In addition, we further compare the performance of window attention, global attention, and sliding window attention in terms of accuracy and attention computational complexity, explaining the rationale behind our adoption of window attention.

Experimental results show that the convolution scheme achieves the lowest performance. This is because the scheme struggles to adapt to depth variations and alignment deviations in BEV features, leading to insufficient robustness and decreased accuracy. By introducing the proposed DSF-Module (window attention), the model improves mAP by 0.6%, while the parameter count increases by only 4.8% compared to the convolution scheme, with no negative impact on model efficiency.

Furthermore, the weight generation of DSF-Module does not rely on global context modeling capability but focuses more on the similarity between image and point cloud features. Therefore, compared with using global attention, window attention lags in performance by only 0.1%, while the attention computational cost is reduced from 8398.1 M MACs to 37.3 M MACs, a reduction of approximately 224×. Compared with sliding window attention, window attention maintains comparable performance while reducing attention computation by approximately 3×. In summary, compared with the base convolution scheme, the proposed DSF-Module achieves a 0.6% improvement in mAP with only 4.8% and 5.4% increases in parameters and computational cost, respectively, striking a favorable balance between accuracy and efficiency.

**Figure 8:**
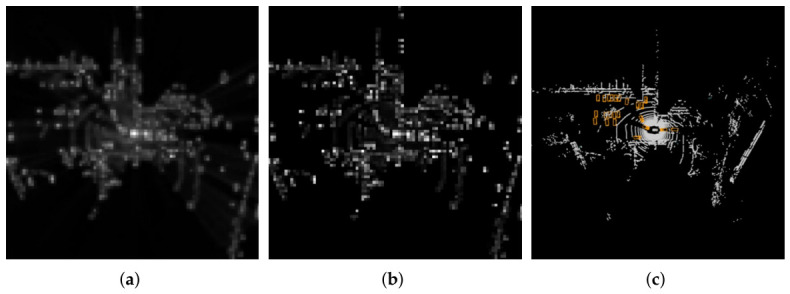
Visualization comparison of multimodal fused features. Figure (**a**) shows the fusion results of the baseline model, Figure (**b**) shows the fusion results after introducing PCGD-Net and DSF-Module, and Figure (**c**) shows the ground-truth point cloud of the scene.

We also visualize the original fused features and the feature representations enhanced by PCGD-Net and DSF-Module. As shown in Figure 8, the left panel shows the baseline fused features, the middle panel shows the fused features from our BEV-Nexus model, and the right panel shows the corresponding ground-truth point cloud scene. It can be observed that the feature representations after our depth perception enhancement and dynamic adaptive fusion are clearer and more discriminative, significantly reducing the misalignment and feature blurring issues caused by simple concatenation.

#### 4.4.3. Ablation Study on DAEB

As shown in Table 9, we conduct an ablation study on DAEB, mainly comparing the impact of dilated convolution and other attention modules on performance. The baseline (without DAEB) achieves 62.8% mAP and 66.8% NDS. Experimental results show that introducing various attention modules consistently improves performance. For example, combining dilated convolution with SE-Block yields improvements of 0.2% in mAP and 0.4% in NDS, while combining it with CBAM yields 0.3% and 0.4%, respectively. Since both they are non-3D attention mechanisms, the performance improvements are limited. When dilated convolution is combined with SimAM (DAEB), the model achieves the best performance of 63.2% mAP and 67.6% NDS, improving over the baseline by 0.4% and 0.8%, respectively. Compared with dilated convolution combined with other attention mechanisms, DAEB does not show a significant advantage in mAP improvement, but achieves a notable gain in NDS.

This result suggests that the energy function and 3D weights adopted by SimAM can more effectively capture spatial relationships. The SimAM preserves the spatial structure of feature maps more faithfully and enables fine-grained 3D weighting across channel, height, and width dimensions. Thus, the performance improvement brought by SimAM not only enhances the overall detection capability (mAP) but also further improves the detection quality (NDS).

## 5. Conclusions

To address the issues of inaccurate image depth estimation and static fusion paradigm in BEVFusion, a BEV perception model named BEV-Nexus is proposed in this paper. First, PCGD-Net is designed to improve the quality of image BEV feature generation, where the depth estimation capability of the image branch is enhanced through depth prior injection, ground-truth loss constraints, and point-cloud-projected depth filling during inference. Second, DSF-Module is proposed to measure the similarity between image and point cloud features via window attention, enabling dynamic and adaptive feature fusion. In addition, DAEB is developed to combine dilated convolution with a parameter-free 3D attention mechanism, further improving the overall feature representation and detection quality. Experimental results on the nuScenes dataset demonstrate that BEV-Nexus achieves 1.8% and 1.5% improvements in mAP and NDS, respectively, over the baseline. Meanwhile, all the above modules are designed to be lightweight, introducing no significant inference time overhead.

BEV-Nexus achieves promising detection performance on the nuScenes dataset and demonstrates steady improvements in horizontal comparisons. However, due to the hardware limitation of a single RTX4090 GPU, experiments with state-of-the-art resolution settings (e.g., voxel size of 0.075 m, image resolution of 704 × 256) were not conducted. Instead, all experiments were carried out under lower-resolution configurations (voxel size of 0.15 m and four-fold downsampled images), where the feature map size is only one-quarter of that in the original BEVFusion, leaving further room for improvement in detection accuracy. In addition, cross-dataset generalization experiments and robustness tests have not been completed due to training time constraints. Future work will focus on performance optimization under low-resolution conditions, cross-dataset validation, and model robustness evaluation.

## Figures and Tables

**Table 1:** Summary of mainstream 3D object detection methods categorized by modality and paradigm.

Modality	Paradigm	Representative Works	Advantages	Disadvantages
Image-based	BEV-based	BEVDet [11], BEVDepth [12]	Unified BEV; multi-task friendly.	Inaccurate depth prediction; complex calculation process.
	Non-BEV	DETR3D [13], PETR [14]	End to end detection; no projection errors.	Poor multi-task friendliness; complex training process.
Point cloud-based	Point-based	PointNet++ [15], PointRCNN [16]	Fine structure retained; high classification accuracy.	Low computational efficiency; sampling algorithm constraints.
	Voxel-based	VoxelNet [17], SECOND [18]	Efficient BEV convolution; structured features.	Small-object degradation; high information loss.
	Others	PV-RCNN [19], PointFormer [20]	Hybrid or attention-based; strong performance.	High complexity; slower inference.
Image-Point cloud fusion	BEV-based	BEVFusion [7,8], SparseFusion [21]	Multi-task friendly; flexible cross-modal interaction.	Depth prediction bias; high BEV grid pooling latency.
	Non-BEV	CMT [22], FUTR3D [23]	End-to-end detection; no explicit projection.	Limited cross-modal interaction; high training difficulty.

**Table 2:** Performance comparison on the nuScenes dataset, Where C and L represent the use of image data or point cloud data, respectively, and ↑ indicates better performance with increasing values of the metric.

Model	Modality	Resolution	Voxel Size	Backbone	mAP ↑	NDS ↑
Validation dataset						
DETR3D [13]	C	1600 × 900	-	Res101	0.303	0.374
PETR [14]	C	1600 × 900	-	Res101	0.370	0.442
BEVFusion_C [7]	C	704 × 256	-	Swin-T	0.356	0.412
BEVFusion_L [7]	L	-	(0.15, 0.15)	VoxelNet	0.583	0.646
CenterPoint [49]	L	-	(0.15, 0.15)	VoxelNet	0.572	0.614
3D-CVF [50]	C&L	-	(0.05, 0.05)	Res18&SECOND	0.527	0.623
FUTR3D [23]	C&L	800 × 450	(0.15, 0.15)	Res101&VoxelNet	0.605	0.653
Transfusion [40]	C&L	800 × 448	(0.15, 0.15)	Res50&VoxelNet	0.618	0.665
BEVFusion [7]	C&L	704 × 256	(0.15, 0.15)	Swin-T&VoxelNet	0.614	0.661
Ours	C&L	704 × 256	(0.15, 0.15)	Swin-T&VoxelNet	0.632	0.676
Test dataset						
BEVFusion [7]	C&L	704 × 256	(0.15, 0.15)	Swin-T&VoxelNet	0.622	0.667
Ours	C&L	704 × 256	(0.15, 0.15)	Swin-T&VoxelNet	0.638	0.681

**Table 3:** Comparison of detection performance across categories on the nuScenes validation set, where Δ denotes the difference in detection accuracy.

Model	Car	Truck	Construction Vehicle	Bus	Trailer	Pedestrian	Motor	Bicycle	Traffic Cone	Barrier
Base	0.864	0.582	0.289	0.747	0.400	0.835	0.679	0.5444	0.706	0.499
Ours	0.872	0.593	0.329	0.755	0.403	0.845	0.679	0.5556	0.732	0.552
Δ	+0.8%	+1.1%	+4.0%	+0.8%	+0.3%	+1.0%	0.0%	+1.2%	+2.6%	+5.3%

**Table 4:** Comparison of error evaluation metrics on nuScenes dataset, where ↑ indicates better performance with decreasing values of the metric, ↓ indicates better performance with decreasing values of the metric and Δ denotes the difference in detection error.

Model	mAP ↑	NDS ↑	mATE ↓	mASE ↓	mAOE ↓	mAVE ↓	mAAE ↓
Baseline	0.614	0.661	0.337	0.261	0.356	0.313	0.192
Ours	0.632	0.676	0.330	0.259	0.317	0.301	0.193
Δ	+1.8%	+1.5%	−0.7%	−0.2%	−3.9%	−1.2%	+0.1%

**Table 5:** Comparison of model complexity, where C and L denote the use of image data and point cloud data, respectively. ↑ indicates that performance improves as the metric increases, while ↓ indicates that performance improves as the metric decreases.

Model	Modal	mAP ↑	NDS ↑	Memory (Mb) ↓	FPS ↑
CenterPoint [49]	L	0.572	0.634	1942	17.7
3D-CVF [50]	C&L	0.527	0.623	4716	8.5
FUTR3D [23]	C&L	0.605	0.653	5210	4.1
Transfusion [40]	C&L	0.618	0.665	4937	7.8
BEVFusion-L [7]	L	0.583	0.646	1693	15.6
BEVFusion [7]	C&L	0.614	0.661	5416	10.1
Ours	C&L	0.632	0.676	5581	10.0

**Table 6:** Ablation results of the overall model, where ✓ means using the module and ↑ indicates better performance with increasing values of the metric.

PCGD-Net	DSF-Module	DAEB	mAP ↑	NDS ↑
-	-	-	0.614	0.661
✓	-	-	0.622	0.665
-	✓	-	0.617	0.663
-	-	✓	0.617	0.667
✓	✓	-	0.628	0.668
✓	✓	✓	0.632	0.676

**Table 7:** Results of PCGD-Net ablation experiment, where PC denotes point cloud and GT denotes ground truth. ↑ indicates better performance with increasing values of the metric. ✓ indicates the use of the module.

PC Spatial Prior	GT Loss Constraint	GT Depth Filling	mAP ↑	NDS ↑
-	-	-	0.614	0.661
✓	-	-	0.616	0.662
✓	✓	-	0.621	0.665
✓	✓	✓	0.622	0.665

**Table 8:** Results of DSF-Module ablation experiment, where ✓ means using the scheme and ↑ indicates better performance with increasing values of the metric.

Conv Scheme	Window Attn	Global Attn	Sliding Window Attn	mAP ↑	Params	AttnMACs
✓	-	-	-	0.622	774 k	-
-	✓	-	-	0.628	812 k	37.3 M
-	-	✓	-	0.629	812 k	8398.1 M
-	-	-	✓	0.628	812 k	149.3 M

**Table 9:** Results of DAEB ablation experiment, where ✓ means using the module and ↑ indicates better performance with increasing values of the metric.

Dilated Convolution	Attention Module	mAP ↑	NDS ↑
-	-	0.628	0.668
✓	SE-Block [47]	0.630	0.671
✓	CBAM [46]	0.632	0.672
-	SimAM [10]	0.629	0.673
✓	SimAM [10]	0.632	0.676

## Data Availability

The nuScenes dataset (version v1.0) used in this study is publicly accessible at https://www.nuscenes.org (accessed on 20 May 2025). All data preprocessing and annotation parsing were performed using the MMDetection3D (v1.3.0) codebase, following its standard data preparation pipeline for nuScenes. The experimental data are contained within the article.
